# A higher proportion of men than of women fainted in the phase without nitroglycerin in tilt-induced vasovagal syncope

**DOI:** 10.1007/s10286-020-00666-5

**Published:** 2020-01-18

**Authors:** Maryam Ghariq, Roland D. Thijs, L. Martine Bek, Erik W. van Zwet, David G. Benditt, J. Gert van Dijk

**Affiliations:** 1 Department of Neurology, Leiden University Medical Centre, K5-Q, PO Box 9600, 2300 RC Leiden, The Netherlands; 2 Stichting Epilepsie Instellingen Nederland (SEIN), Heemstede, The Netherlands; 3 Department of Biomedical Data Sciences, Leiden University Medical Centre, Leiden, The Netherlands; 4 Department of Cardiovascular Medicine, University of Minnesota, Minneapolis, USA

**Keywords:** Tilt table testing, Vasovagal syncope, Nitroglycerin, Sex, Fainting

## Abstract

**Purpose:**

Vasovagal syncope (VVS) affects more women than men. We determined whether this sex ratio affects tilt table test (TTT) results.

**Methods:**

We retrospectively studied TTT outcomes in suspected VVS. TTT consisted of supine rest, a maximum 20 min of head-up tilt without and, if nitroglycerin was needed, a further maximum 20 min after nitroglycerin administration. TTT was terminated if VVS occurred. We used binary logistic regression for the entire TTT and for each phase, with VVS as outcome and age and sex as predictors.

**Results:**

TTT provoked vasovagal (pre)syncope in 494 out of 766 tests (64%). The proportion of men and women who fainted during the entire TTT did not differ significantly between the sexes (*p* = 0.13, corrected for age). A lower proportion of women than men had VVS in the phase without nitroglycerin (odds ratio 0.54; 95% confidence interval 0.37–0.79; *p* = 0.002, corrected for age), whereas a higher proportion of women than men fainted after nitroglycerin (odds ratio 1.58; 95% confidence interval 1.13–2.21; *p* = 0.008, corrected for age). These sex differences remained significant after correction for a history of orthostatic versus emotional triggers. The effect of sex on TTT outcome was closely associated with differences of blood pressure change upon tilt-up (lower in men in both TTT phases: without nitroglycerin *p* = 0.003; with nitroglycerin *p* = 0.05), but not with heart rate changes.

**Conclusion:**

Men were more susceptible to induction of VVS without nitroglycerin and women after it. The unexpected findings may be due to sex-specific pathophysiological differences.

**Electronic supplementary material:**

The online version of this article (10.1007/s10286-020-00666-5) contains supplementary material, which is available to authorized users.

## Introduction

Syncope is a form of transient loss of consciousness (TLOC) that is due to global self-terminating cerebral hypoperfusion; it is characterized by a rapid onset, short duration and a spontaneous and complete recovery [1]. Vasovagal syncope (VVS) is by far the most frequent cause of TLOC, affecting up to 40% of the general population [2, 3]. VVS is classically triggered by orthostatic stress and/or emotional triggers such as pain, venipuncture or the sight of blood.

VVS appears to exhibit a bimodal age distribution with peaks during adolescence and after the age of 60 [2–5]; further, it is reported to affect women about 1.5 times more frequently than men [6–9]. The reasons for sex-related difference of susceptibility are for the most part not understood [10, 11].

Orthostatic stress is used to assess susceptibility to VVS during tilt table testing (TTT). TTTs consist of supine rest followed by head-up tilt, without the use of nitroglycerin (NTG). In some protocols this ‘no-NTG phase’ is followed by an ‘NTG phase’ [12, 13]. NTG is a potent vasodilator that is reported to increase the sensitivity of the test, although with some loss of specificity [14, 15].

In a data subset we noticed that more men than women fainted in the no-NTG phase of TTT. This observation was unexpected for two reasons. Firstly, the literature does not suggest that interactions of sex and NTG affect the yield of TTT. Secondly, it might be expected that men, generally less prone to VVS, would require a stronger provocation to trigger VVS, such as NTG use. If so, they should have a low tendency to faint without NTG.

We were concerned that our initial observation of greater male susceptibility to non-NTG induced VVS may have been due to a selection bias. Consequently, we initiated this study to assess in a larger population undergoing TTT for suspected VVS, whether susceptibility to fainting during TTT differed between men and women depended on the test phase (no-NTG and NTG). Our secondary aim was to explore possible explanations of sex differences of TTT outcomes by studying certain features of the patient’s clinical data (particularly orthostatic versus emotional triggers or both) and the initial hemodynamic response to tilt-up.

## Methods

We selected all TTTs performed between January 1, 2011, and December 31, 2017, at the tertiary syncope outpatient clinic of the Leiden University Medical Centre. The first inclusion step was to select TTT undertaken due to a clinical suspicion of VVS as the reason to order TTT.

The Leiden TTT protocol for VVS has been described previously [16, 17]. In short, we use a modified Italian protocol with 10 min of supine rest, a no-NTG phase of 20 min at 60–70 degrees of head-up tilt, followed by sublingual administration of 400 µg of NTG and another 20 min of head-up tilt. The protocol routinely comprises continuous blood pressure (BP) measurement with either a Finometer^®^ (Finapres Medical Systems, Amsterdam, the Netherlands) or a Nexfin^®^ (BMEye, The Hague, the Netherlands), a one channel electrocardiogram (ECG), video and electroencephalography (EEG). A neurology resident and a technician have continuous access to all signals during the test. TTT is terminated when (1) the allotted time has passed, (2) when frank syncope occurs, (3) when asystole or slowing of the electroencephalogram (EEG) is observed, or (4) when presyncope occurs in conjunction with a patient complaint of being unwell, together with an accelerating blood pressure decrease [18]. As the study was restricted to retrospective anonymous data gathered exclusively in the context of patient care; Dutch law did not require permission by the institutional review board.

Tilt-induced reflex syncope was defined as described previously, using hemodynamic changes, the EEG and a video record [16]. BP had to decrease with a latency after head-up tilt, and BP had to show an accelerating decrease before syncope. We use the term ‘syncope’ when the hemodynamic changes were accompanied by both EEG slowing and clinical signs of loss of consciousness (in a small minority of cases such clinical signs are ambiguous, and then the EEG is used to provide evidence of cerebral dysfunction) [19]. We use the term ‘presyncope’ for the combination of a similar hemodynamic pattern without EEG changes or clinical loss of consciousness. In this study both presyncope and syncope counted as a positive TTT.

We included all subjects with a clinical suspicion of VVS and TTT who showed either tilt-induced vasovagal presyncope/syncope, or no abnormalities at all. Any other diagnosis during TTT led to exclusion, such as psychogenic pseudosyncope, orthostatic hypotension or the Postural Orthostatic Tachycardia Syndrome. Violations of the test protocol, missing TTT data or artifacts that hampered judgment also led to exclusion.

We gathered clinical data for all eligible subjects, obtained during an outpatient visit before TTT. We recorded the clinical diagnosis, age, sex, a history of cardiovascular disease, movement disorders, hypertension, diabetes mellitus, polyneuropathy, and current use of anti-hypertensive drugs, antipsychotic drugs or antidepressants. In addition, to explore the possible role of triggers in VVS, we recorded if the case notes contained data whether spontaneous VVS had been triggered by either orthostatic stress or emotional issues, including instrumentation and pain, or both. Absence of this information was noted as missing data.

Since we suspected that the hemodynamic response to head-up tilt might predict VVS, we measured the initial HR and BP responses to head-up tilt from a printout of the TTT report. We estimated average HR and BP from 5 to 2 min before head-up tilt (baseline supine), and from 2 to 5 min after head-up tilt (early tilt). HR and BP differences were determined between these two time periods and investigated as to whether these changes contributed to the subsequent positivity of the test.

We defined three outcomes per sex: positive TTT before or after NTG, and negative TTT.

### Statistical analysis

Results are presented as mean ± SD for continuous variables with a normal distribution, as medians with interquartile range for not normally distributed data and as percentages for categorical variables. We used the Mann–Whitney *U* test and Chi-square test where applicable.

We performed a binary logistic regression for the entire TTT and for the different phases with the occurrence of VVS as outcome, and age and sex as predictors. We first restricted analysis to the no-NTG phase, and secondly analyzed the NTG-phase. We corrected for the use of medication by adding this as a predictor in the analyses. Hence, the test compares the sex ratio of those who fainted in a specific phase with the sex ratio of all those entering that phase, while adjusting the result for confounding effects.

To explore potential sex differences we performed an additional analysis by adding the presence of orthostatic trigger and emotional trigger as covariates. To test whether the effect of sex on the outcome would be mediated through BP and HR we used Sobel’s test. ‘Mediation’ here means that BP and HR may influence TTT outcome, while BP and HR are themselves influenced by sex. All analyses were performed using IBM SPSS Statistics version 23 (IBM Corp., Armonk, NY). Statistical significance was set at *p* < 0.05.

## Results

### Participants

**Fig. 1:**
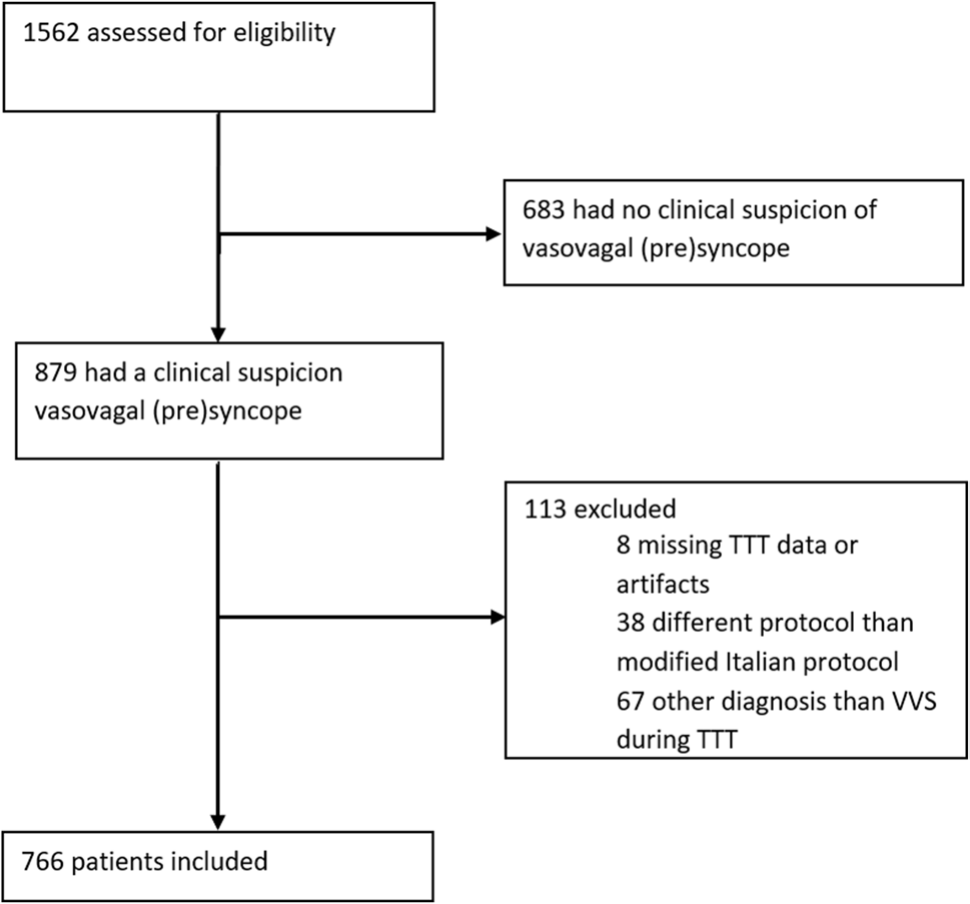
Flow chart of data selection. 1562 cases were assessed for eligibility. 796 cases were excluded, leaving a total of 766 inclusions

Between January 2011 and December 2017, 1562 TTTs were performed to study TLOC or orthostatic intolerance. In 879 cases the reason for ordering TTT was a clinical suspicion of vasovagal syncope (VVS). Of these tests, 113 were excluded for various reasons (i.e. premature termination for reasons other than VVS, or artifacts), leaving 766 cases (Fig. 1).

### Sex, age and TTT

The test population comprised 473 women (62%) and 293 men. As a group, women (median age 36 years, range 10–89 years) were younger than men (median age 51 years, age range 10–87) (*p* < 0.001). Of all women and men included in this study, 66% of the women (*n* = 311) and 62% of the men (*n* = 183) had a positive test (OR 1.27; 95% Cl 0.93–1.74; *p* = 0.13, corrected for age) (Table 1).

**Table 1:** Patient characteristics

	Men (*n* = 293)	Women (*n* = 473)	*p* value
Age, years (median, IQR)	51 (31–62)	36 (21–57)	*p* < 0.001 (MWU)
History	*n* = 292	*n* = 496	
No medical history	40 (13.7%)	75 (16.0%)	*p* = 0.39 (Chi-square)
Hypertension	57 (19.5%)	70 (14.9%)	*p* = 0.10 (Chi-square)
Cardiovascular disease	67 (22.9%)	70 (14.9%)	*P* < 0.01 (Chi-square)
Diabetes mellitus	17 (5.8%)	14 (3.0%)	*p* = 0.05 (Chi-square)
Polyneuropathy	7 (2.4%)	7 (1.4%)	*p* = 0.37 (Chi-square)
Movement disorder	1 (0.3%)	1 (0.2%)	*p* = 0.73 (Chi-Square)
Medication	*n* = 292	*n* = 469	
Antihypertensive	88 (30.2%)	88 (18.8%)	*p* < 0.01 (Chi-square)
Antipsychotic or antidepressant	21 (7.2%)	41 (8.7%)	*p* = 0.48 (Chi-square)

*IQR *interquartile range, *MWU *Mann–Whitney *U* test

In the No-NTG phase a lower proportion of women than men had a positive test: specifically, 14% of all women and 23% of all men had a positive test (OR 0.54; 95% CI 0.37–0.79; *p* = 0.002), corrected for age at time of TTT (age effect *p* = 0.624). In other words, the proportion of sexes who fainted in the no-NTG phase differed from that of those entering that phase, taking age effects into account. Conversely, in the NTG phase, 52% of all women had a positive test in comparison to 40% of all men (OR 1.58; 95% CI 1.13–2.21; *p* = 0.008).

**Fig. 2:**
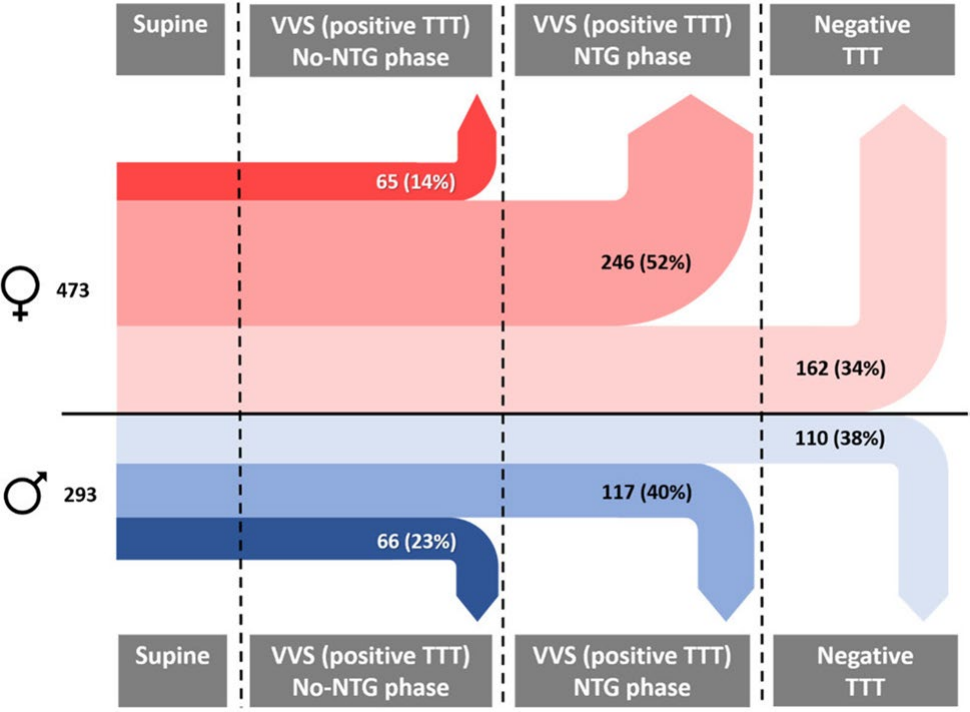
Sex ratio of tilt-induced VVS during the different phases of TTT (rounded percentages). Firstly, the sex ratio during No-NTG phase was analyzed (*n* = 766); more men had a VVS than women (*p* < 0.01). Of all the men and women entering the NTG-phase (*n* = 635), a higher proportion of women fainted than men (*p* < 0.01). The proportion of men and women who fainted during the entire TTT did not differ significantly between the sexes (*p* = 0.13)

A positive test after NTG occurred slightly more frequently in younger patients (OR 0.99; 95% CI 0.98–0.99; *p* < 0.001) (Fig. 2). The use of antihypertensive medication was adjusted for in the analysis; however, the adjustment did not affect the outcome of the results.

### Impact of triggers on tilt response

We attempted to determine if the observed sex differences could be explained by whether spontaneous VVS in individual patients had been preferentially provoked by orthostatic or emotional triggers. The latter information proved inadequate for analysis in 43% of subjects. However, when the spontaneous trigger information was adequate, including the presence of these triggers as covariates did not negate the statistical significance of sex effects. The corrected OR for the No-NTG phase was 0.51; 95% Cl 0.34–0.75; *p* = 0.001 and the corrected OR for the NTG phase was 1.55; 95% Cl 1.10–2.18; *p* = 0.01. (Supplementary Appendix 1).

### Contribution of initial circulatory response after tilt-up to test outcomes

The effect of sex on the occurrence of VVS in the No-NTG phase was closely associated with the nature of the BP change after head-up tilt (*b* =  −0.159, *p* = 0.003, Sobel’s test): patients with a positive response during this phase had a lower increase of BP upon tilt-up. HR had no mediating influence (Table 2).

**Table 2:** Blood pressure (BP) and heart rate (HR) per patient group before and 2–5 min after tilt-up and the change in BP and HR upon tilt

	BP before (mmHg)	BP after tilt-up (mmHg)	BP change (after − before)	HR before (bpm)	HR after (bpm)	HR change (bpm)
Men: VVS No-NTG phase (*n* = 66)	121 ± 21	119 ± 23	−2.0 ± 18	70 ± 14	84 ± 16	14 ± 10
Women: VVS No-NTG phase (*n* = 65)	123 ± 20	123 ± 20	0 ± 15	72 ± 14	90 ± 20	18 ± 1
Men: VVS NTG phase (*n* = 117)	118 ± 19	124 ± 19	5 ± 13	67 ± 10	79 ± 14	12 ± 10
Women: VVS NTG phase (*n* = 246)	122 ± 22	130 ± 22	8 ± 14	72 ± 12	84 ± 15	12 ± 9
Men: Negative TTT (*n* = 110)	118 ± 16	125 ± 20	7 ± 14	68 ± 12	81 ± 15	13 ± 9
Women: Negative TTT (*n* = 162)	121 ± 21	132 ± 23	12 ± 14	72 ± 11	83 ± 14	11 ± 9

Before: Systolic BP 5 to 2 min before tilt-up (mmHg). After tilt-up: Systolic BP 2–5 min after tilt-up (mmHg). BP change: BP after tilt-up minus BP before tilt-up. HR before: HR 5 to 2 min before tilt-up (bpm). HR after: HR 2–5 min after tilt-up (bpm). HR change: HR after minus HR before

The NTG phase showed similar mediating effects of BP (*b* =  −0.054, *p* = 0.05). Again, those with a positive response had a lower BP rise upon head-up tilt. However, the area under the curve was 0.585, showing that this finding is not useful for diagnostic work-up. HR again had no effect (Table 2).

## Discussion

The main finding of this study was that the sex ratios differed between TTT phases among patients undergoing TTT for evaluation of suspected spontaneous VVS. Relatively more men fainted in the drug-free TTT phase: 23% of all men fainted in this phase, in contrast to 14% of all women. This male preponderance was unexpected given the seeming preponderance of women in reporting spontaneous VVS versus men. We had anticipated that men would require a greater stimulus during TTT to induce VVS than would women, but this proved not to be the case, suggesting that there may be as yet unidentified factors leading to systematic pathophysiological differences between fainting under natural circumstances in men and women.

### Previous literature

Sex differences in terms of TTT outcome have not been highlighted as such in the TTT literature. A literature search revealed two papers allowing tabulation of TTT results according to sex similar to our approach. One study [20] on 90 subjects did not demonstrate a significant sex difference. The male-to-female ratio of positive results was 15:20 for the No-NTG phase and 12:26 for the NTG phase. In another study [21] the male-to-female ratios were 32:20 for the No-NTG phase and 42:53 for the NTG phase (*p* = 0.044). In that study, as in ours, men fainted relatively more often in the No-NTG phase and women relatively more often in the NTG phase. These latter two cited studies [20, 21] differed from ours in that in both, a higher percentage of women who entered the test fainted, whereas in our study the percentage of men and women who fainted overall during the TTT did not differ.

### Exploration of possible explanations

Differences in body composition between men and women might in part account for differences in TTT sensitivity in the absence or presence of NTG, and thereby tie in with current ideas regarding the pathophysiology of VVS. Specifically, current concepts point to excessive venous pooling with diminished venous return to the heart causing reduced stroke volume as the step ending in a marked reduction of arterial blood pressure [22]. One possible cause of excessive venous pooling in women might be the relative dose-to-body size effect of NTG: we used a fixed dose, which amounts to a relatively greater concentration in women, who typically have smaller body mass than do men. However, the NTG effect can only contribute to a high rate of VVS in women after NTG; it cannot explain the higher rate of men fainting before NTG. Another potential explanation resides in men having a relatively greater muscle mass than women [23]. If, in VVS-susceptible males, muscles act as a low-compliance vascular bed in which venous blood may pool, then a relatively larger muscle mass will allow more pooling. There is evidence to support the idea that men have a higher rate of venous pooling in the legs than women: in healthy subjects, leg volume increased more in the first 5 min of TTT in men than in women [24]. Unfortunately, there appear to be no data for longer periods of head-up tilt [24, 25]. If men indeed have a higher rate of venous pooling, they may be expected to reach a critical level of excessive pooling earlier than women, i.e., before NTG administration. However, if pooling in muscle mass is to explain their tendency to faint, then this mechanism should also be active after NTG, so men should also faint more readily than women in that phase, the opposite of what we found. Note that similar reasoning might be added for other vascular beds: either a differential sensitivity of the splanchnic and muscular beds to pooling, or a different size of these beds, or a combination of sensitivity and size, might explain sex differences.

Emotional triggers might explain sex differences. Unfortunately, the number of observations was limited, so these data can only be regarded as exploratory. Our findings did not demonstrate sex differences in regard to emotional triggers. However, there are reports that emotional triggers for VVS differ between men and women. Romme et al. examined the influence of age and gender on the occurrence and presentation of reflex syncope and found that emotions/pain and prolonged standing were reported similarly often by men and women as triggers for VVS. Venipuncture was, however, reported the most by women [26]. This was in contrast to the findings of Deveau et al. who, in a post-hoc analysis of the Prevention of Syncope Trial I and II, reported that venipuncture triggered VVS more often in men than in women [27]. In our study selection bias might have occurred due to the tertiary nature of the outpatient clinic, so this should be addressed in future studies.

Lastly, we observed that the blood pressure of patients who fainted without NTG provocation hardly rose shortly after head-up tilt, regardless of sex. In contrast, blood pressure of those who fainted after NTG, or of those who did not faint at all, increased more after head-up tilt. It is likely that those with a low standing blood pressure are more likely to faint earlier. Of note, the orthostatic increase in blood pressure was larger in women directly after TTT, which seems to contradict their overall presumed greater fainting tendency. Thus, our observations suggest that the better blood pressure response to TTT of women makes them more resistant to VVS in the early phases of TTT than men.

More women than men fainted after the application of NTG. NTG is a potent vasodilator that can provoke not only vasovagal syncope but also migraine attacks. Of note, migraine affects more women than men, [28, 29] and VVS is more prevalent in those with migraine, especially in women with migraine [30]. This suggests that female sex hormones might interact with NTG effects on vasodilation. More pathophysiological studies are warranted to study this phenomenon.

## Limitations

The study comprised a relatively large number of patients who underwent a stable TTT-protocol, in whom the presence or absence of (pre)syncope was confirmed unequivocally using video-EEG and finger plethysmography. Nonetheless, several limitations affect the interpretation of the findings. First, the cohort was highly selected by being referred to a tertiary care center and may consequently not represent typical VVS patients. While it is possible that referring doctors refer more men or more women, which we cannot control, it is not clear how this should influence results after referral. Our protocols do not differentiate between men or women at all. Second, while we tried to assess differences between emotional triggers and other causes of spontaneous faints, such distinctions remain uncertain. Thus, whether tilt-induced postural stress reliably identifies outcomes in individuals with non-postural VVS triggers is unproven. Finally, while the association between blood pressure changes at tilt-onset and VVS susceptibility is interesting, a mechanistic connection is uncertain and requires further study.

Although the proportion of men and women who had a tilt-induced VVS during the entire TTT was similar, men proved to be more susceptible to faint without NTG, while a female preponderance was seen in the NTG phase. This was unexpected, as the No-NTG phase most closely resembles the natural situation in which women faint more often than men. In view of these results it is surprising that studies on the pathophysiology of VVS commonly do not explicitly investigate differences between the sexes, let alone interactions of sex and NTG.

## Electronic supplementary material

Below is the link to the electronic supplementary material.

Supplementary file1 (DOCX 30 kb)

## Abbreviations

BP: Blood pressure
HR: Heart rate
LUMC: Leiden University Medical Centre
NTG: Nitroglycerin
OR: Odds ratio
TLOC: Transient loss of consciousness
TTT: Tilt table testing
VVS: Vasovagal syncope
