# Supplementary material for: A higher proportion of men than of women fainted in the phase without nitroglycerin in tilt-induced vasovagal syncope
Source: Clin Auton Res. 2020 Jan 18;30(5):441–7. doi: 10.1007/s10286-020-00666-5 (PMC7561535; doi:10.1007/s10286-020-00666-5)
Supplement: Supplementary file 1 — Supplementary file1 (DOCX 30 kb) [file 10286_2020_666_MOESM1_ESM.docx]

**APPENDIX 1.**

|  | **Orthostatic triggers** | **Emotional**  **triggers** | **Emotional & orthostatic triggers** | **No data or no self-reported trigger** |
| --- | --- | --- | --- | --- |
| **Men: VVS No-NTG phase** | 20 (30%) | 12 (18%) | 10 (15%) | 24 (36%) |
| **Women: VVS No-NTG phase** | 18 (28%) | 11 (17%) | 13 (20%) | 23 (35%) |
| **Men: VVS NTG phase** | 19 (16%) | 23 (20%) | 13 (11%) | 62 (53%) |
| **Women: VVS NTG phase** | 68 (28%) | 33 (13%) | 51 (21%) | 94 (38%) |
| **Men: Negative TTT** | 22 (20%) | 19 (17%) | 11 (10%) | 58 (53%) |
| **Women: Negative TTT** | 33 (20%) | 26 (16%) | 35 (22%) | 68 (42%) |

***Supplementary table 1.*** *Exploration of triggers per patient group.*

| **Trigger for VVS** | **Men (n=293)** | **Women (n=473)** |
| --- | --- | --- |
| - **Only orthostatic triggers** | 61 (21%) | 119 (25%) |
| - **Only emotional triggers** | 54 (18%) | 70 (15%) |
| - **Emotional & orthostatic triggers** | 34 (12%) | 99 (21%) |
| - **No data or no self-reported triggers** | 144 (49%) | 185 (39%) |

***Supplementary table 2.*** *Presence of triggers in men and women.*
